# All-Trans Retinoic Acid Enhances Matrix Metalloproteinase 2 Expression and Secretion in Human Myeloid Leukemia THP-1 Cells

**DOI:** 10.1155/2018/5971080

**Published:** 2018-08-26

**Authors:** Hien Thi Vu, Thi Xoan Hoang, Jae Young Kim

**Affiliations:** Department of Life Science, Gachon University, Seongnam, Kyeonggi-Do 461-701, Republic of Korea

## Abstract

All-trans retinoic acid (ATRA) is an effective drug for the induction therapy of acute promyelocytic leukemia. However, the treatment is associated with adverse events such as retinoic acid syndrome (RAS) in some patients, whose histologic characteristics included organ infiltration by leukemic cells. Matrix metalloproteinase 2 (MMP-2) is often upregulated in tumor cells and plays a role in tumor cell migration and invasion by degrading the extracellular matrix. In this study, we examined the possible modulatory effects of ATRA on MMP-2 expression and secretion in human myeloid leukemia cell line THP-1. The cells were treated with various concentrations of ATRA, and MMP-2 expression and secretion were examined. MMP-2 expression and secretion started to increase with ATRA concentration as low as 0.1 nM and gradually increased thereafter. Agonists of retinoic acid receptor (RAR) or retinoid X receptor (RXR) alone could enhance MMP-2 secretion, and RAR or RXR antagonists alone could reverse ATRA-induced MMP-2 secretion. ATRA increased intracellular calcium ion levels, and a calcium-channel blocker inhibited ATRA-induced MMP-2 secretion. Dexamethasone suppressed ATRA-induced MMP-2 secretion. Our results suggest that ATRA enhances MMP-2 expression and secretion in human myeloid leukemia THP-1 cells in a calcium ion dependent manner through RAR/RXR signaling pathways, and this enhanced expression and secretion may be associated with the possible mechanisms of RAS.

## 1. Introduction

All-trans retinoic acid (ATRA) is the most abundant physiologically active metabolite of vitamin A. It plays important roles in a wide range of biological processes such as the immune response and cell growth, differentiation, and apoptosis [1, 2]. ATRA has been used as an effective drug in the induction treatment of acute promyelocytic leukemia (APL) [3, 4]. APL is characterized by a reciprocal balanced translocation between chromosomes 15 and 17 [5]. This translocation leads to the fusion of the retinoic acid receptor-*α* (RAR*α*) gene and promyelocytic leukemia (PML) gene, resulting in the formation of the PML/RAR-*α* fusion protein, which is involved in leukemogenesis [6, 7]. The therapeutic effect of ATRA is characterized by the degradation of the PML/RAR*α* oncoprotein and differentiation of the malignant cells into phenotypically mature myeloid cells [8]. In addition to its therapeutic usefulness in APL, ATRA has recently attracted great attention for the treatment of other cancers because of its antiproliferative and proapoptotic properties [9].

Matrix metalloproteinases (MMPs) are a family of zinc-dependent endopeptidase, which may be secreted, membrane-bound, or intracellularly located [10]. They are involved in the physiological and pathological remodeling of the extracellular matrix (ECM) by cleaving ECM proteins such as collagen, fibronectin, elastin, and laminin [11]. In addition to their involvement in the normal tissue remodeling, MMPs also act on other substrates to regulate many cellular processes such as cell proliferation, adhesion, migration, apoptosis, chemotaxis, and signaling [11–14]. MMPs are often upregulated in tumor cells and play roles in tumor cell migration and invasion by degrading the ECM [15–18]. Among the 23 MMPs identified in humans [19], MMP-2 is widely expressed in tissues and cells [20]. Recently, the levels of MMP-2 secretion were directly used to evaluate the migration of various types of cells [21, 22].

Previous* in vitro* studies showed that ATRA inhibited MMP-2 expression in human cancer cell lines from glioblastoma [23, 24], breast cancer [25], lung cancer [26], ovarian cancer [27], chondrosarcoma [28], and osteosarcoma [29], but it did not affect [30] or enhance [31] MMP-2 expression in neuroblastoma cell line. However, the role of ATRA in the regulation of MMP-2 expression in myeloid leukemic cells has not been clarified. The purpose of this study was to investigate the possible modulatory effects of ATRA on MMP-2 expression and secretion in human myeloid leukemia THP-1 cells.

## 2. Materials and Methods

### 2.1. Reagents and Chemicals

ATRA, RAR*α* agonist BMS753, RAR*α* antagonist BMS195614, retinoid X receptor (RXR) *α* agonist LG100268, and pan RXR antagonist UVI3003 were purchased from Sigma-Aldrich (St. Louis, MO, USA). Antibiotic-antimycotic, HEPES (4-(2-hydroxyethyl)-1-piperazine ethane sulfonic acid) buffer, and *β*-mercaptoethanol were purchased from Invitrogen Corp (Gibco BRL, MD, USA).

### 2.2. Cell Culture and Treatment

THP-1 cells (Korean Cell Line Bank, Seoul, Korea) were maintained in RPMI 1640 culture medium (Welgene Inc., Daegu, Korea) with 10 mM HEPES buffer and *β*-mercaptoethanol supplemented with 10% heat-inactivated fetal bovine serum (FBS) and 1% antibiotic-antimycotic at 37°C in a 5% CO_2_ humidified incubator. To measure the expression and secretion of MMP-2, the cells were cultured without FBS since it is known to contain large numbers of proteins, including MMP-2 and MMP-9 [32].

### 2.3. Western Blot Analysis

To measure the secreted MMP-2 proteins, the culture supernatants were collected by centrifugation at 400 × g for 5 min, and then concentrated using an Amicon Ultra-15 Centrifugal Filter Unit, 10 kDa cut-off (Millipore Corp., Billerica, MA, USA) at 2000 × g for 20 min. The supernatants were collected and the protein concentrations were measured with the Qubit fluorometer (Invitrogen Corp). Five micrograms of protein was subjected to 10% sodium dodecyl sulfate-polyacrylamide gel electrophoresis and blotted onto a polyvinylidene difluoride (PVDF) membrane (Millipore Corp.). The membrane was blocked with 5% BSA in 1X TBST (2.68 mM KCl, 137 mM NaCl, 25 mM Tris-HCl, and 0.05% Tween 20). Next, the membrane was probed with an anti-MMP-2 (42-5D11, Millipore Corp.) or an anti-*β* actin antibody (Santa Cruz Biotechnology Inc., Dallas, TX, USA). The proteins of interest were detected with an HRP-conjugated goat anti-mouse IgG antibody (sc-2055; Santa Cruz Biotechnology Inc.) or donkey anti-goat IgG antibody (sc-2020; Santa Cruz Biotechnology Inc.) and visualized with ECL-West-Q Pico ECL Solution from GenDEPOT (Barker, TX, USA) using the ChemiDoc MP System (Bio-Rad, Hercules, CA, USA).

### 2.4. Gelatin Zymography

The THP-1 cells were cultured in a serum-free medium for 48 h with or without 1 *μ*M ATRA. The obtained conditioned media were mixed with nonreducing sample buffer and incubated at room temperature for 10 min. The samples were then loaded onto a 10% acrylamide gel containing 0.1% gelatin type A. After electrophoresis, the gel was washed with 2.5% Triton X-100, followed by incubation with developing buffer overnight at 37°C. The gel was then stained with Coomassie brilliant blue and destained with a methanol-acetic acid solution. The gel was visualized on a light box.

### 2.5. Flow Cytometry

To determine the cell surface expression of MMP-2, the cells were first incubated with a purified anti-MMP2 antibody (42-5D11, EMD Millipore Corporation) for 30 min and then with the phycoerythrin-conjugated secondary antibody (Mouse IgG, CLCC35004, Cedarlane Lab, Burlington, Ontario, Canada) at 4°C for 30 min. After washing twice with phosphate buffered saline (PBS), the cells were resuspended in PBS and analyzed on a Cytomics FC500 MLP (Beckman Coulter Inc., Fullerton, CA, USA).

### 2.6. Quantitative Real-Time Polymerase Chain Reaction (qRT-PCR)

To analyze the MMP-2 expression, total RNA was extracted with the Qiagen RNeasy kit (Qiagen, Hilden, Germany) according to the manufacturer's instructions. The RNA concentrations were determined with an SD2000 microspectrophotometer (Bioprince, Atlanta, GA, USA). cDNA was synthesized from 2 *μ*g of total RNA with an MMLV reverse transcriptase (Bioprince) using an oligo dT primer (Bioprince) at 65°C for 1 h. qRT-PCR was performed on the iQ5 multicolor Real-Time PCR detection system using an iQ SYBR Green Supermix (Bio-Rad). PCR amplification was performed using the following primer sets: MMP-2 5′-ttgacggtaaggacggactc-3′, 5′-acttgcagtactccccatcg-3′, *β*-actin 5′-gggacctgactgactacctc-3′, and 5′-agcttctccttaatgtcacgc-3′. The sample was normalized using the human *β*-actin gene as an endogenous control. For each sample, the relative abundance of the target mRNA was calculated from the C^Δt^ values for both the target and endogenous reference gene using the 2^−ΔCt^ cycle threshold method.

### 2.7. Enzyme-Linked Immunosorbent Assay (ELISA)

The secreted MMP-2 was quantified using the Human MMP-2 ELISA Kit (ab100610, Abcam, Cambridge, UK) according to the manufacturer's instruction, and the absorbance was measured with an ELISA Reader (*μ*-Quant, Bio-Tek Instruments, Winooski, USA) at 450 nm.

### 2.8. Intracellular Calcium Measurement

After removing the growth medium, the cells were washed with calcium-free HBSS (Invitrogen) and 20 mM HEPES assay buffer (pH 7.2, Invitrogen). The cells were then mixed with 100 *μ*l of Fluo-4-NW-dye, obtained from Molecular Probes (Invitrogen), and incubated for 30 min at 37°C. Following an additional 30-min incubation at room temperature in the dark, the fluorescence signals were measured using the Cytomics FC500 MLP (Beckman Coulter Inc.) with appropriate for excitation and emission settings of 494 nm and 516 nm, respectively.

### 2.9. Statistical Analysis

All data were presented as mean ± standard deviation (SD), from at least three different experiments. The group means were compared using one-way analysis of variance (ANOVA), followed by post hoc test. All tests were performed using the SPSS statistical software version as mean ± SD. *∗*p < 0.05 was considered statistically significant.

## 3. Results

### 3.1. ATRA Enhances MMP-2 mRNA Expression, but Reduces Cell Surface MMP2 Expression

**Figure 1:**
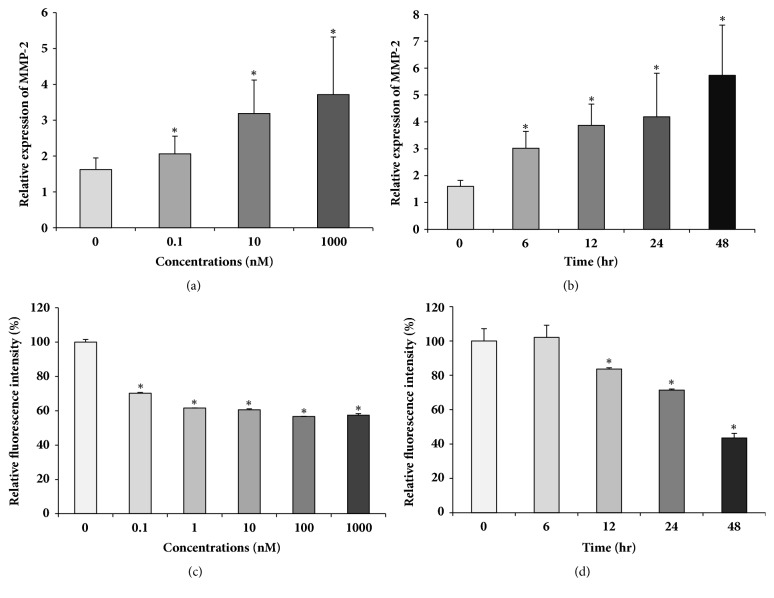
ATRA upregulates MMP-2 mRNA expression but suppresses its cell surface expression of THP-1. THP-1 cells were treated with the different concentrations of ATRA as indicated above for 24 h (a and c) or with 1 *μ*M (b) or 100 nM (d) ATRA for various time points as indicated above. The expression of MMP-2 mRNA was determined by qRT-PCR and was normalized to that of *β*-actin (a and b). The cell surface expression of MMP-2 was analyzed by flow cytometry (c and d). Bar graphs show the relative gene expression ± SD.*∗*P < 0.05 versus DMSO-treated control.

We examined the effects of ATRA on MMP-2 expression in the human myeloid leukemic cell line THP-1. The cells were treated with different concentrations of ATRA, ranging from 0.1 to 1000 nM for 24 h, and the MMP-2 mRNA expression was determined by qRT-PCR. MMP-2 mRNA expression started to increase at ATRA concentration as low as 0.1 nM and peaked (3.7-fold increase) in the presence of 1 *μ*M ATRA (Figure 1(a)). Thereafter, MMP-2 mRNA expression increased gradually, reaching approximately 5 times the control level at 48 h after treatment with 100 nM ATRA (Figure 1(b)). Since it is known that MMP-2 can be membrane-bound or secreted [33], we first examined the cell surface MMP-2 expression using flow cytometry. As shown in Figure 1(c), cell surface MMP-2 expression started to decrease at an ATRA concentration of 0.1 nM, decreasing gradually thereafter, and reaching 60% of control levels after 1 *μ*M ATRA treatment (Figure 1(c)). Cell surface MMP-2 levels started to decrease 12 h after treatment with 100 nM ATRA, decreasing significantly thereafter, and reaching around 50% of the control level at 48 h after the treatment (Figure 1(d)).

### 3.2. ATRA Induces MMP-2 Secretion in Dose- and Time-Dependent Manners

**Figure 2:**
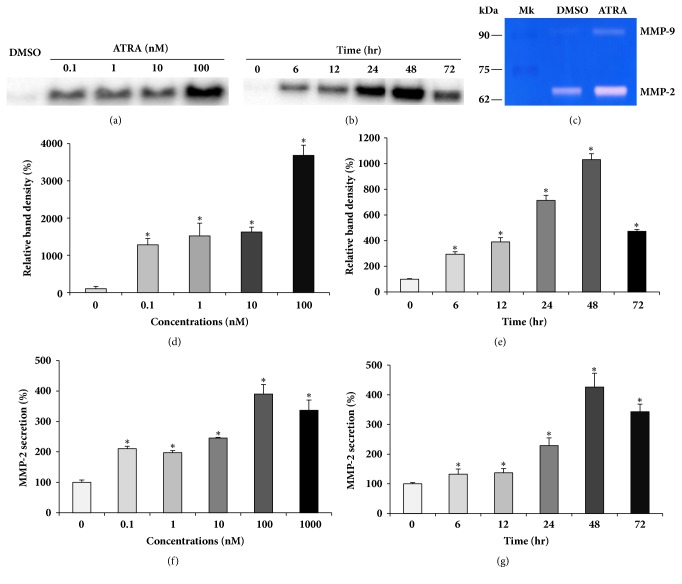
ATRA induces MMP-2 secretion in time- and dose-dependent manners. THP-1 cells were treated with different concentrations of ATRA indicated above (a, d, and f) or with 1 *μ*M ATRA for different time periods indicated above (b, e, and g). The culture supernatants were collected, concentrated by filtration, and subjected to western blot analysis (a and b). The culture supernatants were subjected to ELISA (f and g). Densitometry analysis of MMP-2 bands shown in (a and b) was performed by ChemiDoc MP System (d and e). A representative gel zymogram shows enhanced MMP-2 activity in ATRA-treated cells (c). *∗*P < 0.01 versus DMSO-treated control.

To find the reason behind the reduction of the surface levels of MMP-2 by ATRA, despite MMP-2 mRNA upregulation, we measured MMP-2 secretion with both western blot and ELISA. The cells were treated with various concentrations of ATRA, ranging from 0.1 to 100 nM for 24 h and the culture supernatants were collected, concentrated by filtration, and subjected to western blot analysis. Secreted MMP-2 levels were markedly increased by 0.1 nM ATRA treatment, with a maximum increase of 3700% at 100 nM ATRA concentration (Figures 2(a) and 2(d)). After treatment with 1 *μ*M ATRA, secreted MMP-2 levels gradually increased over time, reaching approximately 1000% of the control level at 48 h, and decreased thereafter (Figures 2(b) and 2(e)). The increase in MMP-2 secretion, measured by western blot, was confirmed by ELISA which revealed similar patterns of increase in ATRA-induced-MMP-2 secretion (Figures 2(f) and 2(g)). To confirm that the secreted MMP-2 was the active form, we performed gelatinase zymography, which is commonly utilized to detect the gelatinolytic enzymatic activities of the active forms of both MMP-2 and MMP-9. As shown in Figure 2(c), the protein band of the MMP-2 active form (molecular weight 68 kD) in the ATRA-treated cells was thicker than that in the DMSO-treated control cells, indicating that ATRA treatment induces the secretion of the active form of MMP-2.

### 3.3. ATRA-Induced MMP-2 Secretion Depends on the RAR/RXR Pathway

**Figure 3:**
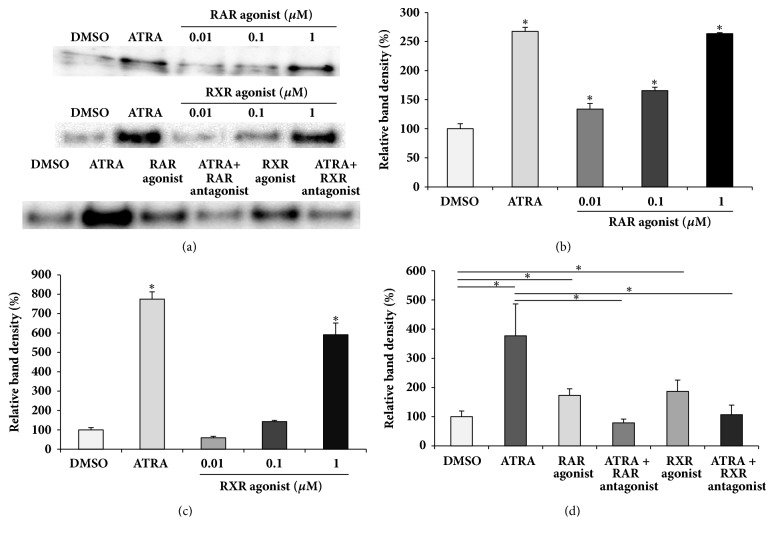
Effect of RAR/RXR agonists or antagonists on MMP2 secretion. Cells were treated with the different concentrations of RAR/RXR agonists indicated above (a, b, and c). Cells were treated with 1 *μ*M RAR/RXR antagonist in the presence of 100 nM ATRA or with 1 *μ*M RAR/RXR agonist without ATRA for 48 h (a and d). The culture supernatants were collected, concentrated by filtration, and subjected to western blot analysis. Protein from each group was loaded at least three times on the gels. Densitometry analysis of MMP-2 bands shown in (a) was performed by ChemiDoc MP System (b, c, and d). *∗*P < 0.01.

Since both RAR and RXR play critical roles in ATRA-regulated gene expression [34], we examined whether MMP-2 secretion is affected by RAR/RXR agonists or antagonists. The cells were treated with different concentrations of RAR/RXR agonist, ranging from 0.01 to 1 *μ*M for 48 h in the absence of ATRA. The RAR agonist induced MMP-2 secretion in a concentration-dependent manner, whereas the effect of RXR agonist on MMP-2 secretion was only pronounced at a concentration of 1 *μ*M (Figures 3(a)–3(c)). The levels of MMP-2 secretion, induced by treatment with 1 *μ*M RAR agonist or RXR agonist, were comparable to those induced by 100 nM ATRA treatment. To assess the effects of RAR/RXR antagonists on ATRA-induced MMP-2 secretion, the cells were treated with 1 *μ*M RAR or RXR antagonist in the presence of 100 nM ATRA for 48 h. As shown in Figures 3(a) and 3(d), the RAR or RXR antagonist completely reversed ATRA-induced MMP-2 secretion.

### 3.4. ATRA-Induced MMP-2 Secretion Depends on Calcium Ions

**Figure 4:**
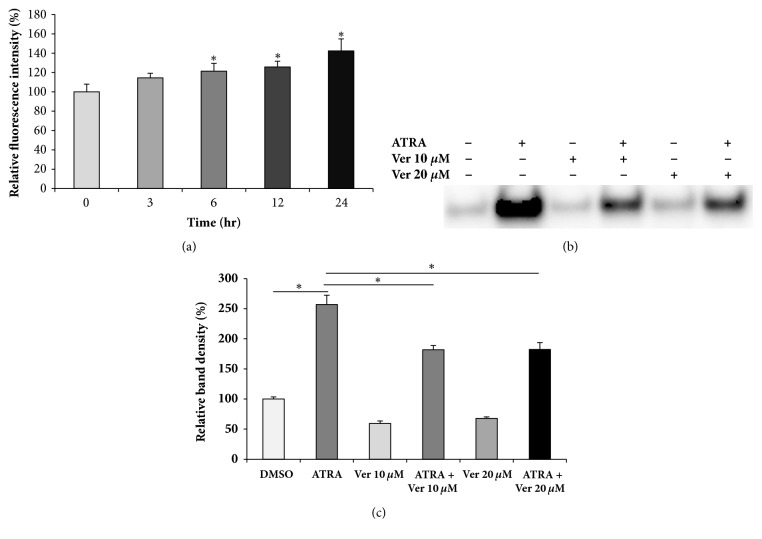
ATRA enhances MMP-2 secretion in a calcium-dependent mechanism. Cells were treated with 100 nM ATRA for various time periods indicated above. Then, cells were collected and intracellular calcium levels were measured by flow cytometry (a). Cells were treated with different concentrations of verapamil (10 or 20 *μ*M) in the presence or absence of 100 nM ATRA for 48 h (b and c). The culture supernatants were collected, concentrated by filtration, and subjected to western blot analysis (b). Densitometry analysis of MMP-2 bands shown in (b) was performed by ChemiDoc MP System (c). *∗*P < 0.05.

Since several studies have suggested the possible relationship between cellular calcium levels and enhanced MMP expression [35–37], we examined the intracellular calcium levels of ATRA-treated cells and performed experiments with calcium-channel blocker to evaluate the possible involvement of calcium ions in ATRA-induced MMP-2 secretion. As shown in Figure 4(a), treatment with ATRA led to a marked increase in the intracellular calcium levels in a time-dependent manner. Verapamil, a calcium-channel blocker, significantly inhibited ATRA-induced MMP-2 secretion (Figures 4(b) and 4(c)), indicating the involvement of calcium ions in ATRA-induced MMP-2 secretion.

### 3.5. Dexamethasone Inhibits ATRA-Induced MMP-2 Secretion

**Figure 5:**
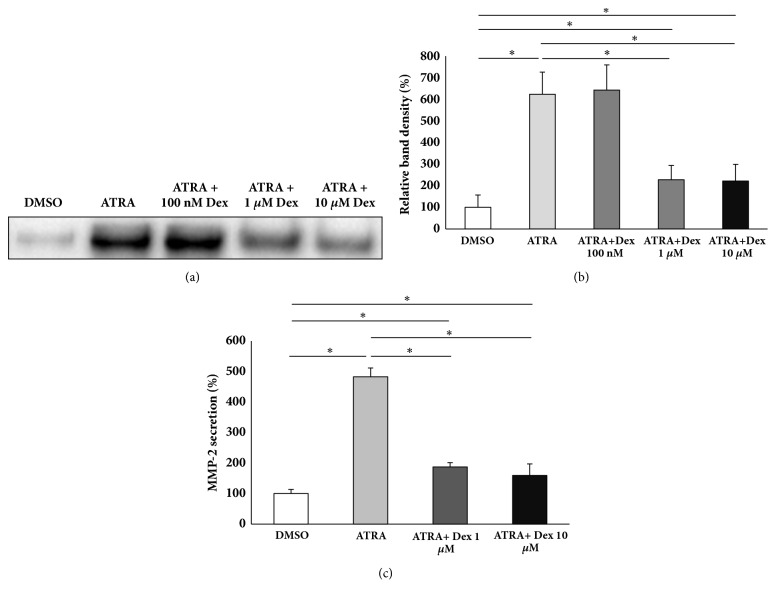
Dexamethasone suppresses ATRA-induced MMP-2 secretion. Cells were pretreated with different concentrations of dexamethasone (Dex) as indicated above for 2 h and were followed by ATRA treatment for 48 h. The culture supernatants were collected, concentrated by filtration, and subjected to western blot analysis (a). Densitometric analysis of MMP-2 bands shown in (a) was performed by ChemiDoc MP System (b). Cells were pretreated with 1 or 10 *μ*M dexamethasone for 2 h and were followed by ATRA treatment for 48 h. The culture supernatants were used for ELISA (c). *∗* p < 0.05.

Since it has been reported that the anti-inflammatory agent dexamethasone inhibits MMP-2 expression [27, 29] and cancer cell migration [38–40], we examined the effect of dexamethasone on ATRA-induced MMP-2 secretion in THP-1 cells. The cells were pretreated with dexamethasone at different concentrations for 2 h, followed by ATRA treatment for 48 h. The culture supernatants were concentrated and subjected to western blot analysis. As shown in Figure 5, 1 *μ*M or 10 *μ*M dexamethasone reversed ATRA-induced MMP-2 secretion, whereas 100 nM dexamethasone had no effect on it (Figures 5(a) and 5(b)). The significant suppression of ATRA-induced MMP-2 secretion, achieved by treating THP-1 cells with 1 *μ*M or 10 *μ*M dexamethasone, was also confirmed by ELISA (Figure 5(c)).

## 4. Discussion

ATRA is known to suppress various types of cancers [41]. The mechanisms of the anticancer effects of ATRA include antiproliferation, proapoptosis, and metastasis inhibition [42]. For tumor invasion and migration, the tumor cells must modulate matrix degradation, cell-cell adhesion, and cell-matrix attachment [43]. MMPs mediate the invasive properties of tumor cells and promote angiogenesis through their ability to degrade basement membranes and remodel the ECM architecture [44–46]. In particular, MMP-2 plays a critical role in tumor cell invasion and metastasis [17, 45–47]. Most previous studies demonstrated that ATRA exerts inhibitory effects on the MMP-2 expression of various tumor types [23–29]. However, our results revealed that ATRA enhances both MMP-2 expression and secretion in human myeloid leukemia THP-1 cells. The reason for the discrepancy between the previous results and our current findings is unknown; however, it may be attributed to the different tumor types used in the studies. Unlike our study, where leukemic cells with indigenous migratory and invasive properties were used, all the previous studies cited above used tumor cells derived from nonleukocytic cells.

Although ATRA is generally well tolerated, development of retinoic acid syndrome (RAS) in some patients has been recognized as a distinct potential life-threatening complication, which is characterized by fever, dyspnea, hypotension, and weight gain [48]. The histologic characteristics of RAS include organ infiltration by leukemic cells [49]. ATRA-induced leukemic cell extravasation was found to be due to the increased adhesion and motility of these cells compared with their undifferentiated counterparts [50–52]. Although the underlying molecular mechanisms for the emigration of ATRA-treated leukemic cells into tissues are yet to be elucidated, this syndrome was effectively treated by administering dexamethasone and by withholding ATRA in severe cases [53–55]. In our study, MMP-2 expression and secretion in THP-1 cells were markedly enhanced by ATRA treatment and pretreatment of THP-1 cells with dexamethasone significantly inhibited ATRA-induced secretion of MMP-2. Since it is known that dexamethasone upregulates glucocorticoid receptor to inhibit NF-*κ*B activity [56], and that ATRA can induce NF-*κ*B activation [57], which results in MMP-2 expression and activation [58], we speculate that inhibitory action of dexamethasone may be due to a suppressive effect on NF-*κ*B activation. Based on our results, we suggest that ATRA-enhanced MMP-2 expression and secretion may be one of the major causes of RAS. However, to verify our hypothesis, further* in vitro* studies with matrigel invasion and transendothelial invasion assays using ATRA, RAR/RXR agonists or antagonists, and MMP-2 selective inhibitors, as well as animal studies, are necessary.

ATRA exerts various biological effects including regulation of gene expression by binding to the nuclear RARs [59, 60]. ATRA binds to RAR to form heterodimers with RXR. The RAR-RXR protein complex binds to retinoic acid response elements (RAREs) or retinoid X response elements (RXREs), each of which has a well-defined DNA sequence and induces gene transcription [61]. Our results revealed that the RAR agonist alone could induce MMP-2 secretion, while the RAR antagonist alone could reverse ATRA-induced MMP-2 secretion, suggesting that ATRA-enhanced MMP-2 expression and secretion depend on the RAR signaling pathway. However, since ATRA does not bind to RXR, our finding that the RXR agonist alone is able to induce MMP-2 secretion, which can be reversed by the RXR antagonist alone, is also indicative that MMP-2 secretion is independent of the RAR mechanisms. Since the RXR agonist, LG100268, used in this study, was capable of activating the PPAR*γ*-RXR heterodimers, PPAR*α*-RXR heterodimers [62], and RXR homodimers [63], the MMP-2 secretion could be mediated by the RXR homodimer signaling pathway or PPAR-dependent mechanisms in addition to the RAR signaling pathway.

In this study, ATRA increased the intracellular calcium levels, and the calcium-channel blocker, verapamil, inhibited ATRA-induced MMP-2 secretion, suggesting that calcium influx is necessary for ATRA-induced MMP-2 secretion. Our results agree with that of the previous study, which showed that ATRA increased the intracellular calcium levels of human teratocarcinoma cells [64]. In that study, the authors demonstrated that the increase in intracellular calcium levels of ATRA-treated cells was due to increased expression of calcium channels. Our results are also in line with earlier findings that calcium ions are required to induce MMP-2 expression [65]. In addition, Zhang et al. demonstrated that enhanced levels of intracellular calcium ions and MMP-2 expression are associated with metastasis of lung cancer cells [66]. Taken together, our results suggest that ATRA induces calcium influx, which may be due to enhanced calcium-channel expression, and is involved in the induction of MMP-2 secretion in human myeloid leukemia THP-1 cells. However, the precise mechanisms of the ATRA-induced increase in intracellular calcium levels and MMP-2 secretion must be clarified in future studies.

In summary, our results show, for the first time, that ATRA enhances MMP-2 expression and secretion in human myeloid leukemia THP-1 cells in a calcium ion dependent manner through RAR-dependent and independent signaling pathways, and this enhanced secretion was reversed by dexamethasone treatment. We suggest that this enhanced expression and secretion of MMP-2 induced by ATRA treatment may be associated with the possible mechanisms of RAS.

## Data Availability

The data used to support the findings of this study are available from the corresponding author upon request.

## Abbreviations

ATRA: All-trans retinoic acid
RAR: Retinoic acid receptor
RXR: Retinoid X receptor
MMP: Matrix metalloproteinase
RAS: Retinoic acid syndrome.
